# Self-Esteem and Academic Engagement Among Adolescents: A Moderated Mediation Model

**DOI:** 10.3389/fpsyg.2021.690828

**Published:** 2021-06-03

**Authors:** Ying Zhao, Zeqing Zheng, Chenchen Pan, Lulu Zhou

**Affiliations:** ^1^Mental Health Education Center, Yangzhou University, Yangzhou, China; ^2^School of Psychology, Capital Normal University, Beijing, China; ^3^School of Education, Hengshui University, Hengshui, China; ^4^School of Psychology, Beijing Sport University, Beijing, China

**Keywords:** academic engagement, academic self-efficacy, adolescents, perceived social support, self-esteem

## Abstract

As an important predictor of academic achievement and an effective indicator of learning quality, academic engagement has attracted the attention of researchers. The present study explores the relationship among adolescent self-esteem and academic engagement, the mediating effect of academic self-efficacy, and the moderating effect of perceived social support. Four-hundred and eighty adolescents (*M*_age_ = 14.92) from the Hebei Province of China were recruited to complete anonymous questionnaires. The results show that self-esteem positively predicted adolescent academic engagement through the indirect mediating role of academic self-efficacy, and the percentage of this mediation effect of the total effect was 73.91%. As a second-stage moderator, perceived social support moderated the mediating effect of academic self-efficacy. Specifically, when students felt more perceived social support, the impact of academic self-efficacy on their academic engagement was greater. Our findings suggest that adolescent self-esteem, academic self-efficacy, and perceived social support are key factors that should be considered together to improve adolescent academic engagement. Therefore, parents and school educators should actively guide adolescents to improve their self-esteem and academic self-efficacy. Parents and educators should also construct an effective social support system to improve students’ perceived social support and enhance their academic engagement.

## Introduction

With the development of positive psychology, human strengths and positive psychological qualities have received widespread attention. Researchers have focused on the positive opposite of burnout – “engagement” – which is defined as a positive, fulfilling, work-related state of mind, characterized by vigor, dedication, and absorption (Schaufeli and Bakker, 2004). Academic engagement extends the concept of engagement, and it refers to the degree to which students engage in educational learning tasks (such as school-related coursework and learning activities) in the process of formal education (George, 2009). Existing literature suggests that high academic engagement promotes academic achievement (Johnson and Sinatra, 2013), improves physical and mental health (Wefald and Downey, 2009), enhances students’ school adjustment ability (Wang and Fredricks, 2014), and reduces students’ dropout decisions (Fan and Williams, 2010). On the contrary, low academic engagement among adolescents can lead to academic failure, dropping out of school, drug abuse, juvenile crime, and the increase of negative emotions such as anxiety and depression (Leslie et al., 2010; Li and Lerner, 2011).

Adolescence is a sensitive and critical period of development (Blackwell et al., 2007), during which adolescents bear heavy schoolwork pressure while also adapting to significant physical and psychological changes. Some adolescents often experience recurring negative emotions such as anxiety and depression (Sahin, 2014). In the Chinese education system, the phenomenon of examination-oriented education is serious. The standard of educational evaluation is single which takes score as only standard and much utilitarian awareness on violating nature of education exists in current education (Wang, 2004). Adolescents’ academic performance is regarded as a critical indicator of their ability to learn (Christenson et al., 2012). Researchers have explored the psychological factors (other than classroom teaching and learning methods) that affect academic performance, and this scholarship has concluded that academic engagement can effectively predict students’ current academic performance (Hershberger and Jones, 2018) and also influence their future functional growth (Fredricks et al., 2016).

However, we reviewed the relevant literature and found that the research on academic engagement has focused generally on college students. Specifically, it has focused on the characteristics of the class environment, such as the teacher-student relationship (Yang and Lamb, 2014) and peer relationships (Fredricks et al., 2004), and the characteristics of the family environment, such as family socioeconomic status (Randolph et al., 2006) and family support (Blondal and Adalbjarnardottir, 2014). There has been little research focus on the relationship between individual characteristics and academic engagement (Li and Li, 2021). Self-esteem and self-efficacy have been confirmed to have an impact on academic engagement, but there is no research to confirm the respective contributions of these two factors, or on their combined impact on academic engagement. Moreover, research on the regulating mechanism of academic engagement is sparse. Therefore, it is necessary to explore the influence of the psychological factors that regulate or intervene in the academic engagement of adolescents; to fully consider the supportive resources of family, school, and society; and to put forward a plan to improve adolescent academic engagement that helps adolescents navigate the sensitive and critical period of adolescence more smoothly.

### Self-Esteem and Academic Engagement

Self-esteem is the evaluation of an individual’s beliefs and attitudes toward his or her abilities and values (Rosenberg, 1965). Self-esteem during adolescence tends to be unstable, because of the many changes that occur in the adolescents’ roles and responsibilities. Self-esteem tends to decline in early adolescence and recover in the middle and later stages of adolescence (Trzesniewski et al., 2003). Adolescents with high levels of self-esteem tend to experience positive self-experiences (Peng et al., 2019), high-quality interpersonal relationships (Cameron and Granger, 2019), and better physical and mental health (Li et al., 2010).

As a basic psychological structure, self-esteem can serve as a motivator for academic engagement (Lim and Lee, 2017). Expectancy-value theory suggests that individuals’ positive self-evaluation can predict academic outcomes, such as academic engagement (Fang, 2016). A study by Sirin and Rogers-Sirin (2015) showed that self-esteem affected the fields related to academic engagement, and that there was a significant positive correlation between self-esteem and academic engagement. The research data of Filippello et al. (2019) found that self-esteem can predict a person’s level of academic engagement. Thus, we propose the following hypothesis:

*H1: Self-esteem positively predicts adolescent academic engagement.*

### Academic Self-Efficacy, Self-Esteem, and Academic Engagement

Another term related to academic engagement that has also attracted widespread research attention is academic self-efficacy. Schunk (2003) defined this term as a student’s judgment of his or her ability to complete an academic task. Alivernini and Lucidi (2011) posited that academic self-efficacy reflected students’ cognitive ability in their academic fields and predicted academic achievement. Many studies have shown that academic self-efficacy has an impact on students’ academic engagement (Uçar and Sungur, 2017; Liu et al., 2020). On the one hand, academic self-efficacy affects students’ academic efforts and persistence. Compared with students with low levels of academic self-efficacy, students with high levels of academic self-efficacy commit to higher goals and academic expectations, have stronger resistance to frustration, and demonstrate greater persistence when facing difficulties (Wright et al., 2012). On the other hand, students’ confidence in their academic ability can influence their participation in school activities and learning tasks (Eccles and Wigfield, 2002). Students who are confident in their academic abilities will put more effort into academic tasks, while those who lack self-confidence will be less engaged in their studies and are more likely to give up.

As mentioned in section “Self-Esteem and Academic Engagement,” self-esteem has a significant impact on academic engagement. However, it remains to be further explored how self-esteem influences academic engagement and what internal mechanism drives this relationship. Self-efficacy theory posits that academic self-efficacy is a motivational factor that can induce and maintain adaptive learning behaviors (Ruzek et al., 2016). Whether self-esteem can affect adolescents’ academic engagement through academic self-efficacy is worthy of in-depth discussion.

Self-esteem and self-efficacy are connected but different concepts (Judge and Bono, 2001). Self-esteem is a positive evaluation of one’s value and importance; that is, an individual’s evaluation of “being a person.” Self-efficacy is the speculation and judgment about whether a person can complete a certain task, and it is the evaluation of the individual’s ability to “do things,” based on experiences in specific activities. Previous literature has shown a significant positive correlation between self-esteem and academic self-efficacy (Batool et al., 2017). Students with positive self-esteem have higher levels of academic self-efficacy (Pahlavani et al., 2015). Both self-esteem and academic self-efficacy affect individual academic engagement, and self-esteem is closely related to academic self-efficacy; therefore, we can reasonably assume that academic self-efficacy is likely to play a mediating role between self-esteem and academic engagement. Thus, we hypothesize the following:

*H2: Academic self-efficacy mediates the association between self-esteem and adolescent academic engagement.*

### Perceived Social Support, Academic Self-Efficacy, and Academic Engagement

Although self-esteem may affect adolescents’ academic engagement through academic self-efficacy, this effect varies from person to person. Perceived social support refers to the individual’s feelings and evaluation of the degree of support he or she receives from family, friends, and important others (Zimet et al., 1988). Social learning theory (Bandura, 1977) suggests that others’ guidance, expectations, and support will affect an individual’s self-efficacy. Academic engagement plays an important role in individual development, but it is malleable and does not always occur autonomously. When individuals perceive high levels of external support and expectations, their positive learning motivation can be stimulated (Gettens et al., 2018), and the strength of this learning motivation has an important impact on students’ academic engagement (Liu et al., 2009).

However, the existing literature lacks the exploration of the mechanism of the impact of perceived social support on academic self-efficacy. Existing studies have shown that perceived social support can regulate the relationship between self-efficacy and learning goals (Bagci, 2016): in the case of high levels of perceived social support, students’ self-efficacy can effectively predict learning goals, and the establishment of learning goals is conducive to students’ academic engagement (King et al., 2013). Similar to the studies described above, we expect the following:

*H3: Perceived social support moderates the relationship between academic self-efficacy and academic engagement.*

**Figure 1:**
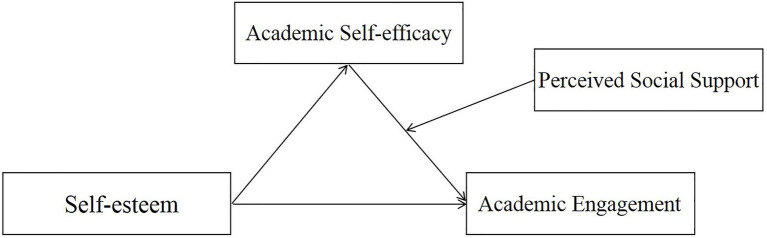
The proposed moderated mediation model.

To sum up, we proposed a moderated mediation model (see Figure 1).

## Materials and Methods

### Participants

The testers were trained in advance to ensure that they fully understood the requirements and precautions of the test. In the present study, the method of cluster sampling was used to invite all of the students of the junior high school grades 7, 8, and 9; all of the students of the senior high school grades 10 and 11; and of two schools in Hebei, China to participate in this study. They were asked to complete the questionnaires anonymously after the informed consent was obtained from the schools, teachers, and parents. Oral informed consent was obtained from each participant, and the participants were permitted to refuse to participate in the study. A total of 520 students voluntarily finished the questionnaires, of which 480 provided valid data (92.31%). Among them, 220 (45.8%) were male students, and 260 (54.2%) were female students. In age, participants ranged from 13 to 17 years, with an average age of 14.92 years (*SD* = 1.47). One-hundred and nineteen students were from grade 7, accounting for 24.8% of the total; 86 students were from grade 8, accounting for 17.9% of the total; 88 students were from grade 9, accounting for 18.3% of the total; 89 students were from grade 10, accounting for 18.5% of the total; and 98 students were from grade 11, accounting for 20.4% of the total. All materials and procedures were approved by the Research Ethics Committee of the corresponding author’s institution.

### Measures

#### Self-Esteem

Self-esteem was assessed using the Rosenberg’s Self-Esteem Scale (RSES; Rosenberg, 1965). This scale consists of a total of 10 items rated on 4-point scales from *strongly disagree* (1) to *strongly agree* (4). The total score can range from 10 to 40, with higher scores representing higher self-esteem. In the present study, the Cronbach’s alpha coefficient was 0.796, indicating an internally reliable scale.

#### Academic Engagement

The Chinese version of the Utrecht Work Engagement Scale for Students (UWES-S; Gan et al., 2007) was used in this study, and the initial version was developed by Schaufeli et al. (2002). The UWES-S is a 17-item scale consisting of three factors: Vigor (six items), Dedication (five items), and Absorption (six items). Participants responded to the items on a 7-point scale from *never* (0) to *every day* (6), with higher scores representing higher levels of engagement. In the present study, the Cronbach’s alpha coefficient of the total scale was high (0.943), and the Cronbach’s alpha coefficients of the three subscales of Vigor, Dedication, and Absorption were 0.846, 0.843, and 0.862, respectively.

#### Perceived Social Support

The Chinese version of the Perceived Social Support Scale (PSSS; Yan and Zheng, 2006) was used in this study, and the initial version was developed by Zimet et al. (1990). The PSSS is a 12-item scale that measures an individual’s subjective perception of social support from family, friends, and others. Participants responded on a 7-point scale ranging from *complete disagreement* (1) to *complete agreement* (7). The total score of the PSSS ranged from 12 to 84, with the higher scores indicating higher levels of perceived social support. The Cronbach’s alpha coefficient for the PSSS in our study was 0.897.

#### Academic Self-Efficacy

The Chinese version of the Academic Self-Efficacy Scale (ASES; Liang, 2000) was used in this study, and the initial version was developed by Pintrich and De Groot (1990). This scale consists of a total of 22 items rated on a 5-point scale from *complete disagreement* (1) to *complete agreement* (5), with higher scores representing greater academic self-efficacy. In the present study, the Cronbach’s alpha coefficient was 0.848.

### Statistical Analysis

Data were analyzed using version 22 of the Statistical Package for the Social Sciences (SPSS) and PROCESS macro 3.3 (Hayes, 2017) in this study. Before the analyses, all continuous variables were mean-centered. First, for all variables, the descriptive statistics and a bivariate correlation analysis were conducted in the SPSS. Then, PROCESS Model 4 (Hayes, 2017) was used to examine the mediating role of academic self-efficacy. Next, regarding the analysis of moderated mediation, a moderated mediation analysis was examined using PROCESS Model 14 (Hayes, 2017). Finally, we conducted a simple slope analysis to test whether the mediation effect of academic self-efficacy was different at different levels of the moderator variable. The dummy coded gender (1 = male and 2 = female) was the control variable in this analysis. Percentile bootstrap confidence intervals were calculated based on 5,000 samples.

### Check for Common Method Bias

This study adopts Harman’s one-factor test (Zhou and Long, 2004) to examine common method biases. Unrotated factor analysis showed that 11 factors were generated and could explain 61.46% of the total variance. The first principal factor explained 23.4% of the variance, which is less than 40%, indicating that there was no serious common method bias in this study.

## Results

### Descriptive Analyses

Table 1 shows the means, SD, and Pearson correlations for all of the variables. Pearson correlation analyses revealed that self-esteem was positively correlated with academic engagement (*r* = 0.23, *p* < 0.01) and academic self-efficacy (*r* = 0.36, *p* < 0.01), and academic engagement was positively correlated with academic self-efficacy (*r* = 0.52, *p* < 0.01).

**Table 1:** Descriptive statistics and correlations among variables.

	1	2	3	4	5	6
1 Gender	—					
2 Age	0.11^*^	—				
3 Self-esteem	0.03	0.01	—			
4 Academic engagement	0.11^*^	−0.24^**^	0.23^**^	—		
5 Academic self-efficacy	−0.05	−0.19^**^	0.36^**^	0.52^**^	—	
6 Perceived social support	0.01	−0.05	0.02	0.01	0.02	—
*M*	1.54	14.92	26.60	84.01	73.29	57.88
*SD*	—	1.47	2.37	18.90	10.66	14.61

*N* = 480.

* *p* < 0.05;

** *p* < 0.01.

### Testing for Mediation

Table 2A shows the mediation analysis results. After controlling for covariates (gender and age), the results showed that in the first step, self-esteem positively predicted academic engagement, *β* = 0.23, *p* < 0.001 (Model 1 in Table 2A). In the second step, self-esteem positively predicted academic self-efficacy, *β* = 0.36, *p* < 0.001 (Model 2 in Table 2A). In the third step, academic self-efficacy positively predicted academic engagement, *β* = 0.47, *p* < 0.001 (Model 3 in Table 2A). Finally, the biased-corrected percentile bootstrap method was used to show that the indirect effect of self-esteem on academic engagement through academic self-efficacy was significant, *ab* = 0.17, *SE* = 0.03, and 95% CI = [0.12, 0.23], the direct effect of self-esteem on academic engagement was not significant, *c*’ = 0.06, *SE* = 0.04, and 95% CI = [−0.03, 0.15], as shown in Table 2B. Therefore, academic self-efficacy fully mediated the relationship between self-esteem and academic engagement. The percentage of this mediation effect of the total effect was 73.91%. These results support Hypotheses 1 and 2 (see Tables 2A and 2B; Figure 1).

**Table 2A:** Mediation effects of academic self-efficacy on the relationship between self-esteem and academic engagement.

Dependent variable	Model 1		Model 2		Model 3	
	*β*	*t*	*β*	*t*	*β*	*t*
Gender	0.25	2.86^**^	−0.08	−1.02	0.29	3.71^***^
Age	−0.17	−6.10^***^	−0.13	−4.84^***^	−0.11	−4.03^***^
Self-esteem	0.23	5.28^***^	0.36	8.10^***^	0.06	1.40
Academic self-efficacy					0.47	8.12^***^
*R*^2^	0.13		0.17		0.31	
*F*	22.01^***^		29.58^***^		41.50^***^	

*N* = 480. Each column is a regression model that predicts the criterion at the top of the column. Gender was dummy coded such that 1 = male and 2 = female.

** *p* < 0.01;

*** *p* < 0.001.

**Table 2B:** The bootstrapping analysis of the mediating effects.

	Effect	*SE*	Boot CI lower	Boot CI upper	Proportion
Total effect	0.23	0.04	0.14	0.32	
Direct effect	0.06	0.04	−0.03	0.15	26.09%
Indirect effect	0.17	0.03	0.12	0.23	73.91%

### Testing for the Moderated Mediation Model

**Figure 2:**
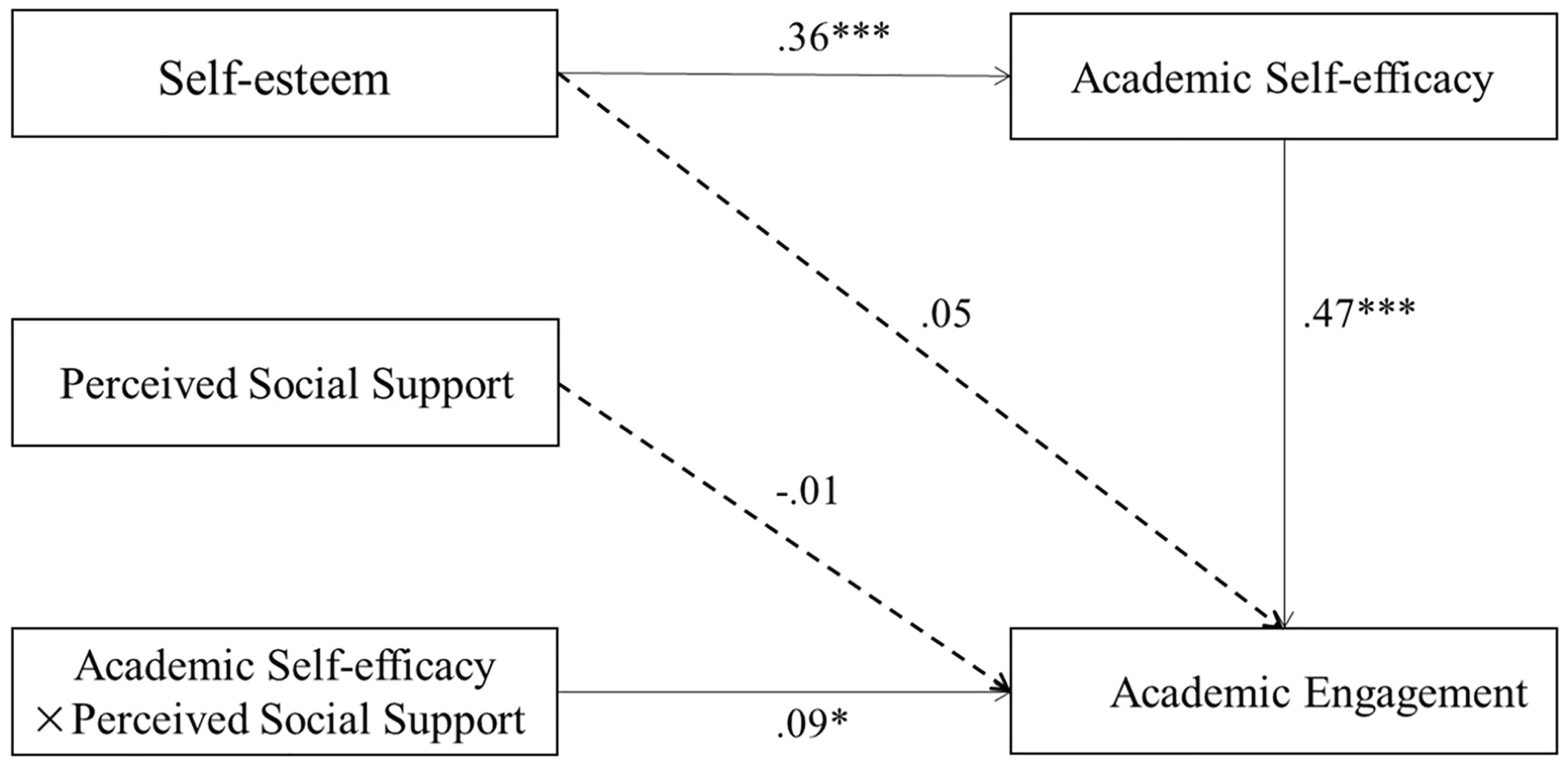
Path coefficients of the moderated mediation model. Covariates were included in the model but are not presented for simplicity. ^*^p < 0.05; and ^***^p < 0.001.

Model 14 of the PROCESS macro (Hayes, 2017) was used to examine the moderating role of perceived social support. Overall testing models are presented in Figure 2, and the specific indirect effects are presented in Table 3A. The results showed that self-esteem positively predicted academic self-efficacy (*β* = 0.36, *p* < 0.001); academic self-efficacy positively predicted academic engagement (*β* = 0.47, *p* < 0.001); self-esteem and perceived social support did not predict academic engagement (*β* = 0.05, *p* > 0.05; *β* = −0.01, *p* > 0.05, respectively); and the interaction effect of academic self-efficacy and perceived social support on academic engagement was significant (*β* = 0.09, *p* < 0.05), and the index of the moderated mediation was 0.03, *SE* = 0.02, 95% CI = [0.01, 0.07], indicating that the association between academic self-efficacy and academic engagement was moderated by perceived social support.

**Table 3A:** Results of perceived social support moderate the mediation process.

Dependent variable	Model 1		Model 2	
	*β*	*t*	*β*	*t*
Gender	−0.09	−1.02	0.30	3.72^***^
Age	−0.13	−4.84^***^	−0.11	−4.04^***^
Self-esteem	0.36	8.10^***^	0.05	1.25
Academic self-efficacy			0.47	8.41^***^
Perceived social support			−0.01	−0.06
Academic self-efficacy × Perceived social support			0.09	1.97^*^
*R^2^*	0.17		0.32	
*F*	29.58^***^		29.76^***^	

* *p* < 0.05,

*** *p* < 0.001.

**Figure 3:**
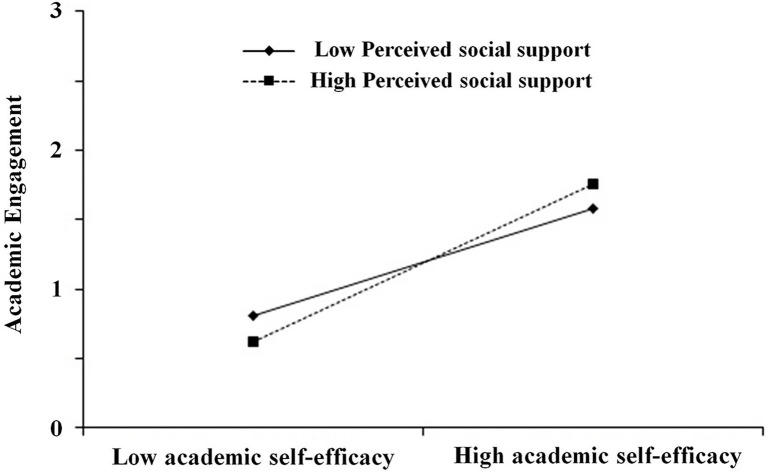
Perceived social support as a moderator on the relationship between academic self-efficacy and academic engagement.

We further conducted a simple slope analysis in SPSS 22.0 to explore the pattern of the moderating effect. Figure 3 presents the perceived social support (M ± SD) as a function of academic self-efficacy and academic engagement. The results indicate that academic self-efficacy was positively correlated with academic engagement for both adolescents with higher perceived social support (*B_simple_* = 0.57, *p* < 0.001) and also for those with lower perceived social support (*B_simple_* = 0.47, *p* < 0.001). Moreover, bias-corrected percentile bootstrap analysis revealed that the indirect effect was more significant for adolescents with higher perceived social support – *β* = 0.21, *SE* = 0.03, 95% CI = [0.14, 0.27] – than for those with lower perceived social support – *β* = 0.14, *SE* = 0.03, 95% CI = [0.07, 0.21], as shown in Table 3B. In sum, these results suggested that perceived social support moderated the relationship between self-esteem and academic engagement *via* academic self-efficacy, supporting Hypothesis 3.

**Table 3B:** Conditional indirect effect of perceived social support when academic self-efficacy mediated between self-esteem and academic engagement.

Mediator	Perceived social support	Effect	*SE*	Boot CI lower	Boot CI upper
Academic self-efficacy	M − SD	0.14	0.03	0.07	0.21
	M	0.17	0.03	0.12	0.23
	M + SD	0.21	0.03	0.14	0.27

## Discussion

The present study investigates the relationship between adolescent self-esteem and academic engagement in order to clarify how the potential mechanism of self-esteem might predict academic engagement. As expected, the results demonstrate (1) a positive association between self-esteem and academic engagement, (2) the mediating effect of academic self-efficacy, and (3) the moderating effect of perceived social support. Moreover, the mediating effect of academic self-efficacy was distinguished as being affected by different levels of perceived social support.

### Self-Esteem and Academic Engagement of Adolescents

The results show that adolescent self-esteem positively predicts academic engagement. High levels of self-esteem can increase the academic engagement of adolescents; these results support our hypothesis and validate the expectancy-value theory. Individuals with high levels of self-esteem set stricter standards and only consider themselves “good enough” when they met those standards, resulting in positive self-evaluation and increasing academic engagement (Filippello et al., 2019). From another perspective, individuals with high levels of self-esteem can effectively alleviate the negative academic emotions caused by high expectations (Kort-Butler and Hagewen, 2011).

The path coefficient between self-esteem and academic engagement was no longer significant after adding the mediating variable (academic self-efficacy), indicating that the influence of self-esteem on academic engagement was entirely through academic self-efficacy. Achievement motivation theory believes that self-esteem can significantly predict individual achievement motivation (Accordino et al., 2000), but there is an inverted U-shaped relationship between motivation level and behavior performance. Only moderate motivation can make individual behavior performance the best. Therefore, self-esteem may not directly predict adolescents’ academic engagement.

### The Mediating Role of Academic Self-Efficacy

This study found that academic self-efficacy played a complete intermediary role between adolescent self-esteem and academic engagement, which verifies our research hypothesis and echoes the research conclusions of other scholars (Pahlavani et al., 2015; Uçar and Sungur, 2017).

The self-esteem level and stability of adolescents are relatively low (Zhang et al., 2010), but most previous studies focused on the self-esteem of other ages, and few studies showed how the self-esteem of adolescents affects their academic engagement. This study shows that adolescent self-esteem does not have a direct effect on academic engagement; rather, it indirectly affects academic engagement through the influence of academic self-efficacy. Students with high self-esteem have higher self-cognition and academic self-efficacy. They can better regulate all aspects of available resources (Ouweneel et al., 2011) and thus achieve their academic expectations and ultimately increase their engagement in learning.

### The Moderating Role of Perceived Social Support

Consistent with our hypotheses, perceived social support moderated the association between academic self-efficacy and academic engagement. Compared with adolescents with a low level of perceived social support, the academic self-efficacy of those with a high level of perceived social support had a more significant predictive effect on academic engagement. Self-efficacy was a stable predictor of individual behavior, and academic engagement was influenced by perceived social support. The predictive effect of self-efficacy on adolescent academic engagement was changed by perceived social support.

Our findings fit with the hypothesis of the “protection factor-protection factor model” (Fergus and Zimmerman, 2005). Academic self-efficacy and perceived social support are both found to be protective factors, and the two promote and strengthen each other. The higher the level of perceived social support, the greater the predictive effect of academic self-efficacy on academic engagement. The results also validated the academic engagement impact model (Skinner and Belmont, 1993), which proposes that the satisfaction of students’ basic psychological needs (autonomy, relatedness, and competence needs) directly influences their academic engagement, and that an external support system affects students’ behavior by satisfying their basic psychological needs. When students establish harmonious and caring interpersonal relationships with surrounding individuals, their relatedness needs can be satisfied, which further stimulates positive behaviors such as hard work, persistence, and active participation (Legault et al., 2006). Similarly, students in classroom situations are more likely to internalize learning motivation and participate in learning activities autonomously when they feel that their basic psychological needs are supported (Niemiec and Ryan, 2009).

This study considered the effect of perceived social support on the relationship between academic self-efficacy and academic engagement from the perspective of interpersonal relationship; however, according to different psychological theories, there may be other factors affecting academic engagement. Family investment theory believes that family socioeconomic status reflects the situation of economic capital, human capital, and social capital in the family environment comprehensively, and affects the learning attitude of students (Randolph et al., 2006). Family socioeconomic status has an impact on academic self-efficacy (Artelt et al., 2003); therefore, family socioeconomic status may also play a moderating role between academic self-efficacy and academic engagement. To sum up, the factors affecting academic engagement should be systematically investigated from different perspectives.

## Limitations and Implications

There are several limitations to this study. First, the cross-sectional survey design used in the present study could not infer or verify the causal relationships among variables; a longitudinal design could be used in future studies. Second, only the self-reporting method was adopted in this study. While our results showed that there was no serious common method deviation, future research should adopt multiple research methods to collect data, such as the interview method and other evaluation methods that involve other actors (teachers, classmates, and parents). Third, due to the limitations of human and financial resources, only students in Hebei Province were selected for the test. Future research will try to sample from all of China and discuss important demographic information about the participants.

Despite the limitations of this study, it has research value and significance. This research explored the relationship among self-esteem, academic self-efficacy, perceived social support, and academic engagement. Previous studies have involved only two or three of these variables; this study used four variables to study and build a reasonable model. This work explored academic self-efficacy plays a mediating role between self-esteem and academic engagement, and it also examines the moderating role of perceived social support, further deepening our understanding of how self-esteem affects academic engagement. The model proposed in this study is helpful for educators to pay more attention to adolescents’ self-esteem, academic self-efficacy, and academic engagement, so as to conduct better psychological intervention for adolescents with insufficient academic engagement.

## Conclusion and Recommendations

This study takes an important step toward investigating the mechanism of the influence of self-esteem on academic engagement by testing a moderated mediation model. Self-esteem may positively predict adolescent academic engagement indirectly through academic self-efficacy. Perceived social support was found to be a second-stage moderator, and the mediating effect of academic self-efficacy between self-esteem and adolescents’ academic engagement was found to be stronger for adolescents with higher levels of perceived social support.

Given these conclusions, we make the following recommendations. First, attention should be paid to the promotion of adolescent self-esteem and academic self-efficacy. Parents and teachers should encourage adolescents to make a positive self-cognition evaluation; they should assist them in setting reasonable learning goals and guide them to reasonable attributions of success and failure when they encounter setbacks. Second, parents and teachers should create a positive and supportive learning environment in which students feel adequately supported, encouraged, and recognized. Peer support groups that use the encouragement given by peers to make students feel part of a community of trust and support should be established. Third, parents and teachers should pay attention to the state of students’ academic engagement and guide adolescents who have low academic engagement, or who seem to be exhibiting weariness and truancy. The teaching design should be novel and interesting, and the teaching method should be suitable for the needs of the students. Discussion and debate can be used to help full engage the students in the class material.

## Data Availability Statement

The raw data supporting the conclusions of this article will be made available by the authors, without undue reservation.

## Author Contributions

LZ contributed to conception and design of the study. YZ performed the statistical analysis and wrote the first draft of the manuscript. LZ, CP, and ZZ revised it critically for important intellectual content. ZZ collected the raw data and organized the database. All authors contributed to the article and approved the submitted version.

### Conflict of Interest

The authors declare that the research was conducted in the absence of any commercial or financial relationships that could be construed as a potential conflict of interest.
